# Mitochondrial haplogroup J associated with higher risk of obesity in the Qatari population

**DOI:** 10.1038/s41598-020-80040-7

**Published:** 2021-01-13

**Authors:** Mohammed Dashti, Hussain Alsaleh, Juan L. Rodriguez-Flores, Muthukrishnan Eaaswarkhanth, Fahd Al-Mulla, Thangavel Alphonse Thanaraj

**Affiliations:** 1 Genetics and Bioinformatics Department, Dasman Diabetes Institute, Kuwait City, Kuwait; 2 Kuwait Identification DNA Laboratory, General Department of Criminal Evidence, Ministry of Interior, Kuwait City, Kuwait; 3 Department of Genetic Medicine, Weill Cornell Medicine, New York, NY 10065 USA

**Keywords:** Computational biology and bioinformatics, Genetics

## Abstract

Obesity, a major risk factor for metabolic disorders, is highly prevalent in Qatari population. Maternal transmission of obesity traits can be significant; for example, X haplogroup is known to be associated with lower BMI and body fat mass in Northern Europeans and T haplogroup which is a sister haplogroup of J is known to be associated with obesity in Caucasian subjects from Austria and Southern Italy. We aimed to delineate the mitochondrial haplogroups and variants associated with obesity in Qatari population. Mitochondrial genomes of 864 Qatari individuals were extracted from whole exome sequencing data with an average coverage of 77X. We distributed the participants into 2 sub-cohorts: obese (BMI ≥ 30) and non-obese (BMI < 30); the mean value of BMI from these two groups were 36.5 ± 5.7 and 26.5 ± 2.6, respectively. Mitochondrial haplogroup profiling followed by uni- and multivariant association tests adjusted for covariates were performed. Qatari individuals with mitochondrial haplogroup J had an increased (twofold) risk of obesity (odds ratio [OR] 1.925; 95% CI 1.234–3.002; *P* = 0.0038; the Bonferroni adjusted *P* value threshold is 0.0041), whereas the individuals with haplogroup X were at low risk of obesity (OR 0.387; 95% CI 0.175–0.857; *P* = 0.019). Further, a set of 38 mitochondrial variants were found to be associated (at P ≤ 0.05) with obesity in models adjusted for age, sex and haplogroup.

## Introduction

Obesity occurs when an individual’s energy balance is positive; i.e., energy intake exceeding energy expenditure leads to weight gain. Obesity is a major risk factor for several chronic diseases, including cardiovascular disorders, diabetes, and cancer. Qatari adults have a high prevalence of obesity, estimated at 49% by the Global Burden of Disease Study^1^ and at 37% by the World Health Organization^2^. Obesity is a multifactorial disease caused by environmental factors, such as a positive energy balance and unhealthy lifestyle decisions, as well as genetic factors, including monogenic and polygenic causes. The interplay among these factors complicates the quantification of the inherited influences on obesity.

Mitochondria have an important role in generating cellular energy via the electron transport chain and oxidative phosphorylation (OXPHOS)^3^. Given that mitochondria play a central role in regulating metabolic flexibility, its dysfunction has been demonstrated in tissues associated with obesity^4,5^. The mitochondria genome is a double-stranded circular molecule of 16.6 kilo base pairs carrying 37 genes that encode for 13 proteins, 22 transfer RNAs, and 2 ribosomal RNAs^6^; the majority of the proteins functioning in the mitochondria are, however, encoded in nuclear DNA. Unlike nuclear DNA, mitochondrial DNA (mtDNA) is maternally transmitted with a restricted repair mechanism, which makes it susceptible to replication errors^7^. The susceptibility rate is increased at the non-coding region of the mitochondria, called the D-loop region, especially under oxidative stress. These mtDNA mismatches lead to accumulated variants, such as single nucleotide polymorphisms (SNPs) and insertions/deletions (INDELs); such variants enable the tracing of maternal lineages. Individuals who share the same clusters of mitochondrial SNPs are assigned to a haplogroup, which is a major branch point in a phylogenetic tree. The effects of mitochondrial haplogroups and both inherited and acquired SNPs on several chronic disorders have been explored^8–10^.

Maternal transmission of obesity has been demonstrated in animal models in which female mice with diet-induced obesity passed down obesity to 3 subsequent generations, despite the offspring being fed a regular diet^11^. In line with the notion that deviations from the physiological number of mtDNA copies are expected to be deleterious, a study showed that the total number of mtDNA copies was correlated with obesity before and after gastric bypass and bariatric surgery^12^. A novel mitochondrial tRNA^Cys^ mutation at MT:5802A > G observed in 3 matrilineal relatives has been attributed as the probable cause for obesity in the family with matrilineally inherited obesity^13^. Furthermore, the mitochondrial haplogroup T has been suggested to increase the risk of obesity in 2 independent association studies on Austrian^14^ and Southern Italian^15^ populations. Maternal haplogroups X and H have been proposed to reduce the risk of obesity in Caucasians of northern European origin in the United States (US)^16^ as well as in Arabs living in Kuwait^17^, respectively. It is worth noting that these studies on maternal haplogroups were performed using variants that were extracted either from genome-wide genotype data generated using BeadChips in genome-wide association studies or from sequencing partial sections of the mitochondria genome using Sanger sequencing. Both genotyping strategies limit the number of analyzed SNPs and subsequently impact the resolution of maternal haplogroup prediction and the overall results. Next-generation sequencing (NGS) offers high throughput for large cohorts while still offering the advantages of faster processing and lower cost compared with Sanger sequencing. A number of studies have demonstrated that both whole genome and exome sequencing can indirectly target the whole mtDNA with good coverage^18,19^, and such studies have addressed the genetic basis of both monogenic^20,21^ and multifactorial disorders^22^.

In this study, we extracted mtDNA from whole exome data on 864 Qatari individuals and evaluated the mitochondrial variants and haplogroups for associations with obesity.

## Results

### Study population

The clinical characteristics of the Qatari samples are presented in Table 1. The mean value of BMI of the individuals from the cohort was 32.7 ± 6.8. The obese and non-obese groups differed significantly in sex distribution (*P* < 0.001), age (*P* = 0.006), and body mass index (BMI) (*P* < 0.001). The mean value of BMI from these two groups were 36.5 ± 5.7 and 26.5 ± 2.6, respectively.

**Table 1:** Clinical characteristics of the Qatari cohort study.

	Obese (N = 532) n (%)	Non-Obese (N = 332) n (%)	Total (N = 864) n (%)	*P*-value^@^
**Sex**				
Male	276 (51.9%)	75 (22.6%)	351 (40.6%)	< 2.2e-12
Female	256 (48.1%)	257 (77.4%)	513 (59.5%)	
**Age (years)**				
26–34	27 (62.8%)	16 (37.2%)	43 (4.98%)	0.006
35–44	122 (70.9%)	50 (29.1%)	172 (19.9%)	
> = 45	383 (59.1%)	266 (40.9%)	649 (75.1%)	
Mean ± SD	51.7 ± 10.9	53.7 ± 10.9	52.5 ± 11	
Median (IQ)	51 (44–59)	54 (47–60)	52.5 (45–59)	
**BMI**^**#**^				
Mean ± SD	36.5 ± 5.7	26.5 ± 2.6	32.7 ± 6.8	< 2.2E-16
Median (IQ)	35.4 (32.1–39.8)	27 (24.9–28.7)	31.6 (28–36.7)	

^**@**^*P*-value for age categories for obese versus non-obese groups were calculated using Mann–Whitney U test. *P*-value for sex counts in obese versus non-obese groups were calculated using Chi-sq test. Abbreviations: BMI, body mass index; N, number of individuals; SD, standard deviation; IQ, inter-quartile.

^**#**^The distribution of the participants onto normal weight (BMI 20 to < 25): overweight (BMI 25 to < 30): obese (BMI 30 to < 40): morbid obese (BMI ≥  = 40) = 88: 224: 253: 280.

### mtDNA coverage and variants

**Figure 1:**
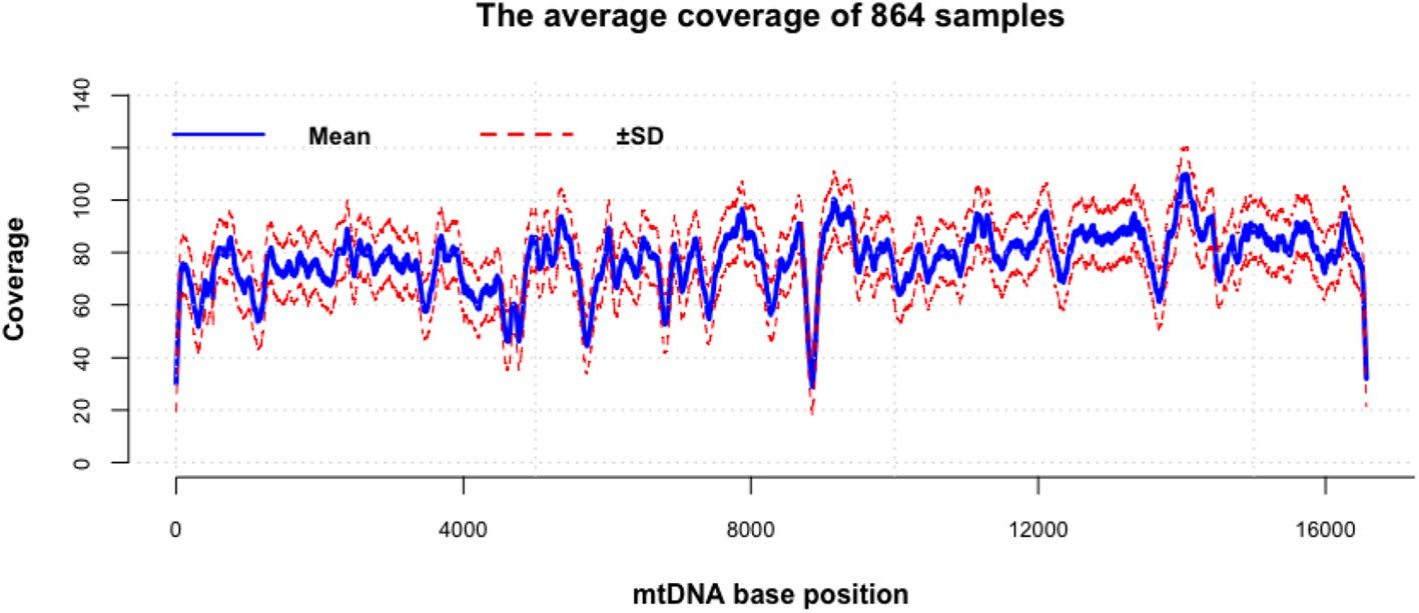
Average coverage of 864 Qatari sample across the whole mtDNA using whole exome sequencing data.

The average coverage across the whole mitochondrial genome of the Qatari whole exome samples was 77X (Fig. 1), while the average coverage of the mtDNA of the Qatari whole genome samples (technical replica) was 4537X.‬‬‬

A total of 1831 mitochondrial variants (including 1775 SNPs and 56 INDELs) were identified using the whole exome samples. All mitochondrial SNPs identified in the 8 whole genomes were captured by the whole exomes, apart from one SNP, namely, MT:3492 A > C, which we identified only in the whole genomes of 4 individuals. Regarding INDELs, we observed inconsistencies between the whole exome and the whole genome in 4 instances (at MT:302, MT:8271, MT:16,179, and MT:16,182).

### Mitochondrial haplogroup association with obesity

**Figure 2:**
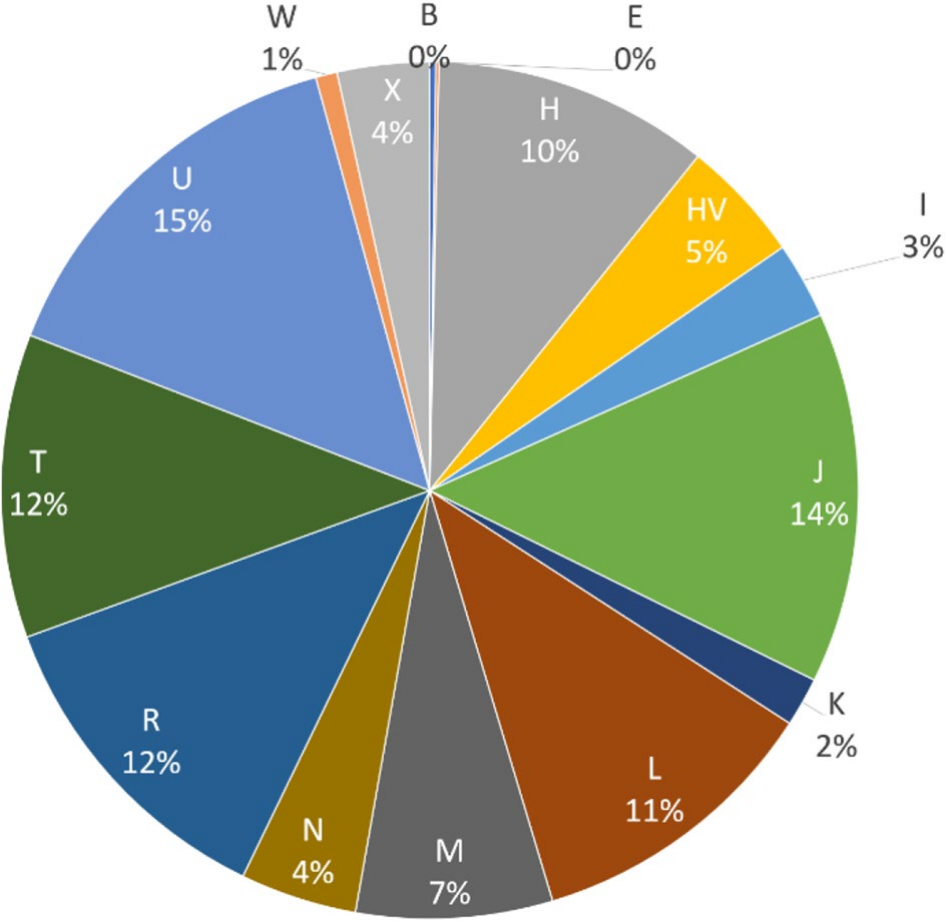
Frequencies of mitochondrial haplogroups observed in the 864 Qatari samples.

The average quality score of predicted haplogroups for all our samples using HaploGrep2 was 93%. In total, we identified 15 major mitochondrial haplogroups (B, E, H, HV, I, J, K, L, M, N, R, T, U, W, and X) in the 864 whole exome samples (Fig. 2). The most common mitochondrial haplogroups in the Qatari samples were U (15%), J (14%), R (12%), T (12%), and L (11%). The mitochondrial haplogroup assignments observed in the 8 whole genomes (technical replica) were the same as those observed in their respective whole exomes (Table 2).

**Table 2:** Mitochondrial haplogroup assignment comparison between whole exome and whole genome.

Exome samples	Exome haplogroups	Genome samples*	Genome haplogroups
SRR5264031	T1a2	SRR2098266	T1a2
SRR5264030	L3e1a1a	SRR2098265	L3e1a1a
SRR5264035	T1a8a	SRR2098260	T1a8a
SRR5264033	X2m'n	SRR2098200	X2m'n
SRR2969965	R0a2c	SRR2969967	R0a2c
SRR5264034	M36a	SRR2098210	M36a
SRR5264029	HV1a3a	SRR2098185	HV1a3a
SRR5264032	L0a2a2a	SRR2098177	L0a2a2a

*Sample ID are NCBI SRA sample number for Qatari whole exome and genome samples.

**Figure 3:**
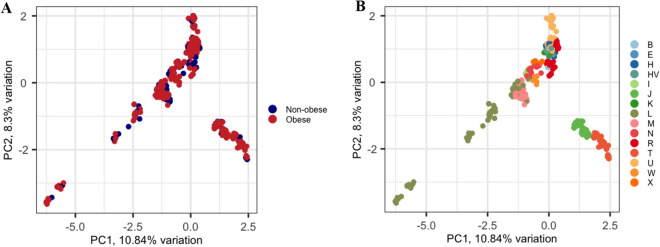
Principal component analysis of the 864 Qatari samples based on their mtDNA. (**A**) Obesity status was considered: the blue color represents the non-obese individuals and the red color represents the obese individuals. (**B**) Haplogroup profiling was considered. PC1 and PC2 on x- and y-axis represent Principal component 1 and Principal component 2 and their variations in percentage, respectively.

Figure 3 shows the clustering patterns of the samples based on principal component analysis, considering obesity status and mtDNA haplogroup. The samples clustered well when we considered the maternal ancestral background through their mtDNA haplogroups rather than considering the obesity status or sex (plot not shown).

**Figure 4:**
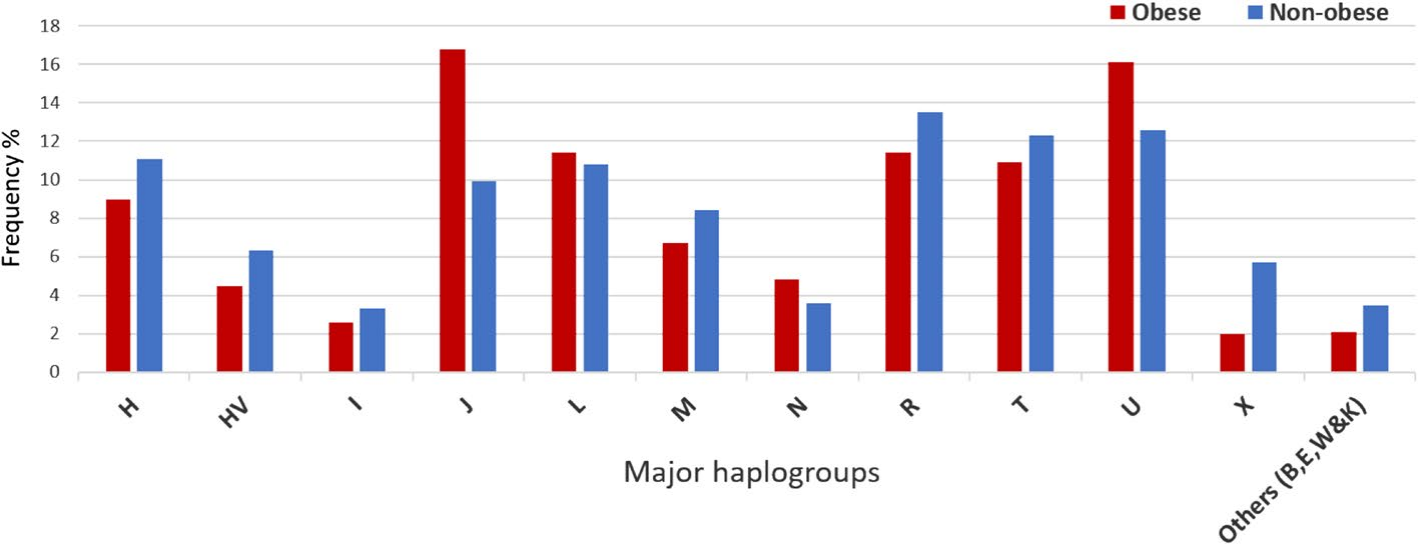
Distribution of major mtDNA haplogroups frequency in obese and non-obese groups.

In mitochondrial comparison analysis, haplogroups with limited number of individuals were grouped together as one group called “others”. Table 3 and Fig. 4 show the mitochondrial haplogroup frequencies for the individuals with obesity and without obesity. The results based on Fisher’s exact test on haplogroup frequencies indicated that individuals with the J haplogroup were at increased risk (approximately twofold) for obesity (OR 1.93, 95% CI 1.234–3.002, *P* ≤ 0.0038; models adjusted for age and sex using multivariate logistic regression); In contrast, individuals with the mitochondrial X haplogroup were at low risk of obesity (OR 0.387, 95% CI 0.175–0.857, *P* = 0.019; models adjusted for age and sex). Applying Bonferroni correction for mitochondrial haplogroup comparisons by dividing *P*-value of 0.05 by 12 (total mtDNA haplogroups) gave a *P-*value threshold corrected for multiple testing of 0.0041 to identify statistically significant associations. Only the J haplogroup has an adjusted *P-*value of 0.0038 (after adjusting for age, sex and haplogroups) which makes it statistically significant against the Bonferroni corrected *P*-value threshold of 0.0041.

**Table 3:** Mitochondrial haplogroups associated with obesity in Qatari population.

Haplogroups	Obese N (532) n (%)	Non-Obese N (332) n (%)	OR (95% CI)*	*P*-value*
H	48 (9%)	37 (11.1%)	0.839 (0.521–1.350)	0.469
HV	24 (4.5%)	21 (6.3%)	0.716 (0.380–1.351)	0.303
I	14 (2.6%)	11 (3.3%)	0.863 (0.373–1.997)	0.731
J	88 (16.8%)	33 (9.9%)	1.925 (1.234–3.002)	** < 0.004**
L	61 (11.4%)	36 (10.8%)	0.998 (0.630–1.582)	0.994
M	36 (6.7%)	28 (8.4%)	0.699 (0.405–1.206)	0.198
N	26 (4.8%)	12 (3.6%)	1.109 (0.530–2.320)	0.784
R	61 (11.4%)	45 (13.5%)	0.823 (0.533–1.271)	0.38
T	58 (10.9%)	41 (12.3%)	0.94 (0.601–1.470)	0.788
U	86 (16.1%)	42 (12.6%)	1.297 (0.855–1.968)	0.221
X	11 (2%)	19 (5.7%)	0.387 (0.175–0.857)	**0.019**
Others (B,E,W&K)	7 (2.1%)	19 (3.5%)	1.606 (0.643–4.012)	0.311

*Values after adjustment for age and sex. Abbreviations: N, number of individuals; OR, odds ratio; CI, confidence intervals of logistic regression test.

### Mitochondrial variant association with obesity

**Figure 5:**
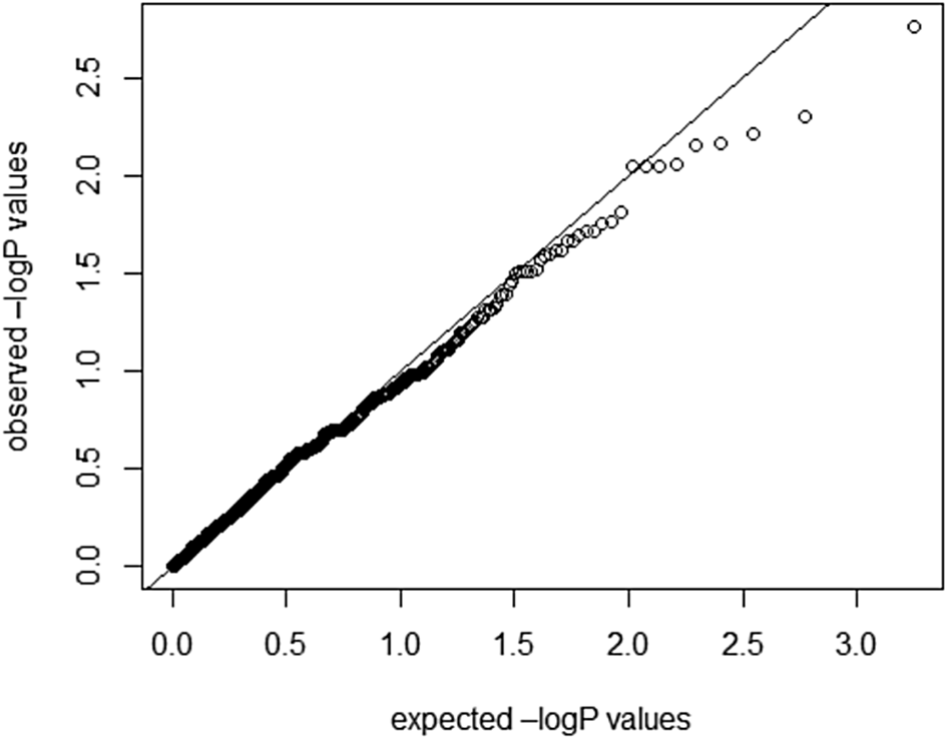
Quantile–quantile plots of the expected and observed − log(*p*-values) for the association of mitochondrial variants with obesity (λ = 1.0035) upon correction for age, sex and haplogroups.

Quantile–quantile plots depicting the expected and observed − log (*p*-values) for association of the mitochondrial variants with obesity are presented in Fig. 5. Genomic-control inflation factor (**λ)** was observed as 1.0035 in tests with corrections for age, sex and haplogroups. The value at close to 1.0 do not necessitate correcting association statistics for genomic-control inflation.

Table 4 presents the results of multiple regression test, performed to identify the variants associated with obesity at *P*-values < 0.05; it is to be noted that we did not apply Bonferroni correction towards multiple testing for total number of variants analyzed (n = 1831) for the *P*-value threshold for significance (= 0.05/1831 = 0.000027]). We observed a set of 38 mtDNA variants to have nominal associations (*P*-value ≤ 0.05) with obesity, with models adjusted for age, sex, and mitochondrial haplogroup. 26 were positively correlated with the risk of obesity, and 12 were negatively correlated with risk of obesity. Of these variants associating with obesity, five (namely, MT:295C > T, MT:11377G > A, MT:12171A > G, MT:16145G > A and MT:16222C > T) were seen correlated with others in the list of associated variants. Conditional analysis using the top leading MT:16069C > T variant with the lowest p-value from Table 4 as the conditioning SNP, identified 14 of the remaining variants remaining as significant. We assume that these 14 variants (see the footnote to Table 4) to have an independent effect on the phenotype with respect to the conditioned SNP. We assume that the remaining 23 mitochondrial variants (that included the above-mentioned LD-dependent 5 variants) from Table 4, which lost their significance, are dependent on the top leading mitochondrial variant.

**Table 4:** Mitochondrial variants associated with obesity in Qatari population.

mtDNA Variants^@^	Gene	Consequence	Frequency in Obese group	Frequency in Non-obese group	Frequency in J haplogroup	Frequency in X haplogroup	OR (95% CI)*	*P*-value *
MT:16069C > T	*TP*	Upstream	0.169	0.099	0	0	2.106 (1.323–3.355)	0.001
MT:295C > T	*RNR1*	Upstream	0.164	0.103	0.004	0	1.948 (1.223–3.103)	0.004
MT:13708G > A	*ND5*	Missense	0.18	0.117	0	0.03	1.849 (1.192–2.869)	0.006
MT:8473 T > C	*ATP8*	Synonymous	0.035	0.006	0.07	0	7.77 (1.753–34.43)	0.006
MT:152 T > C	*RNR1*	Upstream	0.339	0.244	0.42	0.13	1.564 (1.132–2.162)	0.006
MT:4216 T > C	*ND1*	Missense	0.299	0.228	0	0	1.564 (1.12–2.185)	0.008
MT:10499A > G	*ND4L*	Synonymous	0.031	0.006	0.15	0	7.416 (1.649–33.36)	0.009
MT:12570A > G	*ND5*	Synonymous	0.031	0.006	0.15	0	7.416 (1.649–33.36)	0.009
MT:15679A > G	*CYB*	Synonymous	0.031	0.006	0.15	0	7.416 (1.649–33.36)	0.009
MT:11377G > A	*ND4*	Synonymous	0.031	0.009	0.15	0	4.808 (1.351–17.12)	0.015
*MT:15257G* > *A*	*CYB*	*Missense*	*0.037*	*0.015*	*0.19*	*0*	*3.46 (1.245–9.618)*	*0.017*
MT:13966A > G	*ND5*	Missense	0.022	0.063	0	0	0.377 (0.169–0.844)	0.017
MT:14470 T > C	*ND6*	Synonymous	0.02	0.057	0	0	0.363 (0.156–0.847)	0.019
MT:6371C > T	*CO1*	Synonymous	0.02	0.057	0	0	0.363 (0.156–0.847)	0.019
MT:11002A > G	*ND4*	Synonymous	0.035	0.012	0.15	0	3.802 (1.232–11.74)	0.02
MT:750G > A	*RNR1*	Non-coding	0.018	0.054	0	0.26	0.365 (0.155–0.862)	0.021
MT:153A > G	*RNR1*	Upstream	0.02	0.06	0	0.03	0.376 (0.163–0.866)	0.021
*MT:14766 T* > *C*	*CYB*	*Missense*	*0.135*	*0.177*	*0*	*0*	*0.540 (0.315–0.925)*	*0.024*
MT:16051A > G	*TP*	Upstream	0.133	0.087	0.07	0	1.778 (1.078–2.932)	0.024
MT:3010G > A	*RNR2*	Upstream	0.139	0.084	0.19	0	1.776 (1.078–2.924)	0.024
MT:4991G > A	*ND2*	Synonymous	0.041	0.015	0.09	0	3.161 (1.148–8.705)	0.025
*MT:7476C* > *T*	*TS1*	*Non-coding*	*0.035*	*0.015*	*0.19*	*0*	*3.205 (1.144–8.98)*	*0.026*
*MT:11251A* > *G*	*ND4*	*Synonymous*	*0.274*	*0.222*	*0*	*0*	*1.453 (1.035–2.039)*	*0.03*
*MT:15452C* > *A*	*CYB*	*Missense*	*0.274*	*0.222*	*0*	*0*	*1.453 (1.035–2.039)*	*0.03*
*MT:9156A* > *G*	*ATP6*	*Synonymous*	*0.001*	*0.012*	*0*	*0*	*0.082 (0.008–0.789)*	*0.03*
MT:8386C > T	*ATP8*	Synonymous	0.02	0.003	0.09	0	9.874 (1.236–78.87)	0.03
MT:12171A > G	*TH*	Non-coding	0.02	0.003	0.09	0	9.874 (1.236–78.87)	0.03
MT:5501A > G	*ND2*	Synonymous	0.033	0.006	0.16	0	5.238 (1.157–23.72)	0.031
MT:2352 T > C	*RNR2*	Non-coding	0.007	0.027	0	0	0.259 (0.074–0.904)	0.034
MT:14212 T > C	*ND6*	Synonymous	0.005	0.024	0	0	0.223 (0.055–0.908)	0.036
MT:16145G > A	*TP*	Upstream	0.135	0.084	0.32	0	1.674 (1.022–2.741)	0.04
MT:930G > A	*RNR1*	Non-coding	0.005	0.024	0	0	0.231 (0.057–0.939)	0.04
MT:16222C > T	*TP*	Upstream	0.08	0.042	0.46	0	1.984 (1.028–3.83)	0.041
MT:11914G > A	*ND4*	Synonymous	0.11	0.06	0.008	0	1.759 (1.013–3.055)	0.044
*MT:9103 T* > *C*	*ATP6*	*Missense*	*0.016*	*0.003*	*0.06*	*0*	*8.299 (1.021–67.45)*	*0.047*
*MT:5814 T* > *C*	*TC*	*Non-coding*	*0.001*	*0.012*	*0*	*0*	*0.097 (0.009–0.979)*	*0.048*
*MT:15218A* > *G*	*CYB*	*Missense*	*0.011*	*0.03*	*0.008*	*0*	*0.334 (0.112–0.994)*	*0.048*
MT:16261C > T	*TP*	Upstream	0.135	0.084	0.36	0	1.637 (1.004–2.669)	0.048

*Values after adjustment for age, sex and maternal haplogroup; 8 of the listed variants in the table did not attain significant P-values (≤ 0.05) when the model was not adjusted for age, sex and haplogroup—such variants are shown in italics font. Abbreviations: OR, odds ratio; CI, confidence intervals.

^@^Conditional analysis using the top leading MT:16069C > T variant with the lowest p-value as the conditioning SNP, identified 14 of the remaining variants remaining as significant. We assume that these 14 variants to have an independent effect on the phenotype with respect to the conditioned SNP. The 14 mitochondrial variants are: MT:152 T > C, MT:8473 T > C, MT:10499A > G, MT:12570A > G, MT:15679A > G, MT:13966A > G, MT:6371C > T, MT:14470 T > C, MT:750G > A, MT:153A > G, MT:16051A > G, MT:9156A > G, MT:930G > A, MT:11914G > A. We assume that the remaining 23 mitochondrial variants, which lost their significance, are dependent on the top leading mitochondrial variant.

Furthermore, we found 8 additional variants (not included in Table 4) that were detected in only one of the BMI groups in more than 3 individuals. Of these 8 variants, (a) 7 were observed in the groups with obesity, including 5 synonymous variants in *MT-ND4L* (15 individuals), *MT-ND6* (14 individuals), *MT-CO2* (11 individuals), *MT-ND5* (7 individuals), and *MT-ND2* (7 individuals) genes, as well as 2 non-coding variants in *MT-TP* (12 individuals) and *MT-TF* (7 individuals) genes; (b) 1 was observed in the group without obesity, namely, a synonymous variant in the *MT-ND1* gene (4 individuals).

We assessed the clinical significance of the associated variants by examining the ClinVar, Mitomaster, and Mitomap databases and found that none of the variants was annotated as a pathogenic mutation for obesity or for other disorders.

Most of the variants identified as associated with obesity were located within the same mitochondrial genes. However, the mitochondrial genes harboring synonymous variants were only positively associated with the risk of obesity, such as cytochrome c oxidase III (*MT-CO3*), nicotinamide adenine dinucleotide (NADH) dehydrogenase 2 (*MT-ND2*), NADH dehydrogenase 4 (*MT-ND4*), NADH-ubiquinone oxidoreductase chain 4L (*MT-ND4L*), and non-coding variants; as well as tRNA histidine (*MT-TH*), tRNA serine 1 (*MT-TS1*), and tRNA phenylalanine (*MT-TF*). On the other hand, genes harboring both synonymous and missense variants were only negatively associated with the risk of obesity, such as cytochrome c oxidase I (*MT-CO1*) and non-coding variants, tRNA cysteine (*MT-TC*), and tRNA threonine (*MT-TT*).

## Discussion

Our study employed whole exome reads to delineate the mitochondrial DNA variants and haplogroups. The mtDNA coverage from whole exome data sequenced using the Agilent V5 kit is good across the whole genome (Fig. 1). The principal component analysis (PCA) clustered samples of the same haplogroup well. The delineation of mtDNA variants using whole exome reads was consistent when whole genome reads were employed. The only exceptions were inconsistency in calling the MT:3492 and in the length of INDELs at MT:302, MT:8271, MT:16,179, and MT:16,182. Similar SNP-related and INDEL-related alignment errors with NGS data at some of these positions have been reported in earlier studies^23,24^.

Our study of 864 Qatari individuals revealed the following maternal lineage composition for Qatar: 71% Western Eurasian, 11% African, and 12% Asian, figures that agree with the maternal lineage results reported for Arabs in Kuwait^25^, Iraq^26^, and Saudi Arabia^27^.

Our study identified the mitochondrial haplogroup J, the second most frequent haplogroup in the cohort, as significantly associated with a higher risk of obesity. This haplogroup had a significantly higher frequency in the group with obesity than in the group without —obesity. Subclade J1b was the main contributor in our Qatari cohort, with 9% of the samples displaying J1b, a rate similar to the frequency distribution observed in Saudi Arabia (9.4%)^27^. In contrast to our observation of J haplogroup associated with a higher risk of obesity in Qatar, Nardelli et al. (2013) reported that the frequency of haplogroup J was lower in the group with morbid obesity and that the J haplogroup conferred a lower risk of obesity in the Southern Italian population. In a study by Ebner et al. (2015) on Austrian juveniles and adults, haplogroup J showed no association with obesity. These contradictory findings are probably due to differences in geographic origin or simply to differences in study design (such as BMI thresholds for defining obesity status and consideration of only the D-loop region).

Both the above-mentioned studies by Nardelli et al. and Ebner et al. reported that T haplogroup was associated with a risk of obesity in Caucasians from Southern Italy and Austria, respectively, whereas our study showed that J haplogroup was associated with obesity in Arabs from Qatar. It is interesting to note that J and T are sister haplogroups and share polymorphisms^14^, such as MT:4216 T > C, which was significantly correlated with risk of obesity in our study (Table 4). However, the frequency of haplogroup T was not significantly different between our study groups with and without obesity (Fig. 4) and was not associated with obesity (Table 3), which might be due to the fact that the frequencies of haplogroups and their defining variants can vary even among closely related populations, such as Caucasian populations^28^.

The frequency of mitochondrial haplogroup X was higher in the group without obesity than the group with obesity in our study cohort, which is consistent with reported observations that Caucasians of northern European origin in the US with haplogroup X have lower BMI and fat mass values^16^. All the individuals within haplogroup X belonged to Eurasian branch X2, which is present in Saudi Arabia^27^ and Yemen^28^.

A recent study^17^ reported a higher frequency of haplogroup H in the control group (BMI < 25 kg/m^2^) compared with the group with overweight and obesity (BMI > 25 kg/m^2^) among Arabs living in Kuwait. The frequency of haplogroup H in our Qatar study cohort was slightly higher in the group without obesity; nevertheless, the difference was not significant compared with the group with obesity, which might be due to differences in the admixture of Arab ethnicities in the region.

To prioritize our results, we focused on 23 exonic variants that were significantly associated with obesity in the univariate and multivariate analysis (Table 4), as well as 6 exonic variants detected in only 1 of the 2 BMI groups. Most of these variants are within the NADH dehydrogenase subunit genes, complex I, which is involved in cell energy production^24^; as a result, the prioritized variants (as listed in Table 5) in this study can influence their gene function and are associated with body fat mass and obesity^14,29^. *MT-RNR1*, which harbors negatively correlated non-coding variants (MT:930G > A and MT:750G > A), is involved in metabolic homeostasis with protective function against diet-induced obesity^30^. The *MT-RNR2* gene, which harbors negatively associated non-coding variant MT:2352 T > C, is known to protect against oxidative stress^31^. Cytochrome c oxidase subunit I and II genes regulate the OXPHOS system, which is essential for energy production and survival^32^. Another gene that regulates OXPHOS is the ATP synthase protein 8 (*MT-ATP*), where we found 2 synonymous variants (MT:8473T > C and MT:8386C > T). Lastly, mutations within the cytochrome b gene (*MT-CYB*) can cause prominent exercise intolerance, which can lead to obesity^33^. We identified a synonymous variant (MT:15679A > G) in this gene that was positively associated with obesity.

**Table 5:** List of the prioritized variants in NADH dehydrogenase subunit genes which can influence their gene function and are associated with body fat mass and obesity in Qatari population.

Gene	Variant	Impact on encoded protein	Frequency in J haplogroup	Frequency in X haplogroup
MT-ND1	MT:4059C > T	Synonymous	0	0
	MT:4216 T > C	Missense	0	0
MT-ND2	MT:4991G > A	Synonymous	0.09	0
	MT:5501A > G	Synonymous	0.16	0
	MT:5153A > G	Synonymous	0	0
MT-ND4	MT:11914G > A	Synonymous	0.008	0
	MT:11002A > G	Synonymous	0.15	0
	MT:11377G > A	Synonymous	0.15	0
MT-ND4L	MT:10499A > G	Synonymous	0.15	0
	MT:10685G > A	Synonymous	0	0
MT-ND5	MT:13966A > G	Synonymous	0	0
	MT:13708G > A	Synonymous	0	0.03
	MT:12570A > G	Synonymous	0.15	0
	MT:12519 T > C	Synonymous	0	0
MT-ND6	MT:14212 T > C	Synonymous	0	0
	MT:14305G > A	Synonymous	0	0
	MT:14470 T > C	Synonymous	0	0

The mtDNA variants previously associated with obesity were not significant in our study: MT:146T > C, MT:228G > A, MT:263A > G, MT:16294C > T, MT:16296C > T and MT:16526G > A in the Austrian study^14^; MT:4823 and MT:8873 from the study of Caucasians of northern European origin living in the US^16^; and MT:8994G > A from the German and French study^34^.

There are limitations in our study that need to be acknowledged. None of the reported associations involving mitochondrial DNA variants and obesity passes the Bonferroni corrected *P*-value thresholds. The threshold value for BMI (30 kg/m^2^) used to define the obesity and no obesity/control groups differs from that used in previous obesity studies using mtDNA^14–17,29,34–36^. We set a higher threshold for BMI in our study mainly because of the low number of lean BMI individuals in the cohort (the mean BMI value of the cohort was 32.7 ± 6.8 kg/m^2^) and the desire to balance the sample sizes of the 2 groups; however, the mean BMI value in the group without obesity was borderline (26.5 ± 2.6 kg/m^2^), and that of the group with obesity was much higher (at 36.5 ± 5.7 kg/m^2^). Although we used different BMI groupings, we managed to show that haplogroup X was associated with lower BMI, in line with earlier observations^16^. In addition, our study did not explore heteroplasmy, due to the mtDNA coverage and the fact that it would be more suitable with the enriched capture kit for whole mitochondrial genome (higher coverage) and from a variety of specimens.

To conclude, we conducted the largest association tests of mitochondrial haplogroups and variants with obesity performed in the Middle East. Furthermore, our results demonstrated that Qatari individuals with haplogroup J are at increased risk (approximately twofold) of obesity. Our results also confirmed that the frequency of mitochondrial haplogroup X are low in obese individuals in the Qatari population; however, the association signal did not survive the correction for multiple testing.

## Materials and methods

### Ethics statement

This study was reviewed and approved by the institutional Ethical Review Committee at Dasman Diabetes Institute, Kuwait in accordance with the declaration of Helsinki. The human whole-exome data used in this study were publicly available from the National Center for Biotechnology Information Sequence Read Archive. The original studies^37,38^ that generated these data and made available at the public resource had obtained written informed consent of participants who were recruited under protocols approved by the Institutional Review Boards of Hamad Medical Corporation and Weill Cornell Medical College in Qatar.

### Study exome data

Whole exome read data from individuals living in Qatar^37,38^, as sequenced using Agilent SureSelect Human All Exon V5 and V4 kits (Agilent Technologies Inc., USA) on the Illumina HiSeq platform, were publicly available from the National Center for Biotechnology Information Sequence Read Archive (SRA accessions SRP060765, SRP061943 and SRP061463). We downloaded the data and considered only those exomes from native Qatari individuals and those that were sequenced with the Agilent V5 kit (Agilent Technologies Inc., USA), resulting in a data set of 864 native Qatari individuals. We divided this cohort into 2 groups based on their BMI: 532 individuals in the group with obesity (BMI of ≥ 30 kg/m^2^) and 332 in the group without obesity (BMI < 30 kg/m^2^). We downloaded whole genome sequence data on 8 individuals (common with whole exome samples, sharing the same sample identification number) available from the same Qatari studies and used the data to validate the mitochondrial variants called using exome data.

### mtDNA sequences and variant calling

We mapped the raw paired-end reads to human genome reference version GRCh37 using Burrows-Wheeler Aligner version v07-17 with default parameters^39^. We employed the Picard tool version 2.20.2 (http://broadinstitute.github.io/picard) to flag and remove duplicate reads. We employed SAMtools version 0.1.19^40^ to extract the mtDNA reference sequence (NC_012920.1) and the Genome Analysis Tool Kit (GATK) version v3.8-1-0^41^ to calculate the average mtDNA coverage. Subsequently, we created a Genomic Variant Call Format (GVCF) file for each sample using the GATK haplocaller and combined the GVCF files with GATK CombineGVCFs into a single GVCF file for each sequencing capture of whole genome and whole exome samples. We employed GATK GenotypeGVCFs for mtDNA variant identification.

### Haplogroup prediction

The final mtDNA variants were in Variant Calling Format files for both the whole exome and whole genome (technical replica) samples, and these were used for haplogroup profiling, which was achieved by using the HaploGrep 2 tool^42^, based on phylotree build 17 (accessed on 2 February 2020). To assess the accuracy of the mitochondrial haplogroup assignment for a whole exome sample, we compared the results with those obtained using the whole genome data when both the whole exome and genome sequence data were available for the sample.

### Statistical analysis

We used R software (version 3.6.2) (https://www.R-project.org/) to perform the statistical analysis on the clinical characteristics. The descriptive statistics for the categorical variables are presented as numbers and percentages, whereas those for continuous variables are presented as mean ± standard deviation and median and interquartile range. We performed normality tests on the traits (such as age and BMI) using the Shapiro–Wilk test and comparisons between the groups with and without obesity using the nonparametric Mann–Whitney U test. In addition, we applied the chi-squared test to determine whether there was a differential distribution of sex in the BMI groups. We conducted a PCA to test whether the mtDNA clustered the samples based on their BMI groups (obesity and no obesity) and to reveal any other hidden relationships such as haplogroup and sex. We used the PCAtools package on R software to conduct the PCA analysis.

We performed Fisher’s exact tests to examine the associations between the mtDNA haplogroups and the obesity and no obesity groups. We calculated the ORs and 95% confidence intervals (CI) for each haplogroup and set *P* < 0.05 as the threshold for statistical significance. Furthermore, to adjust for age and sex, we performed a logistic regression using IBM SPSS Statistics Version 25 software. To examine the associations for the mtDNA variants between the obesity and no obesity groups, we employed Fisher’s exact test and logistic regression tests from PLINK version 1.9^43^. A 2-tailed *P-*value < 0.05 was considered significant. To adjust for multiple comparisons, we applied Bonferroni correction by dividing *P*-value of 0.05 by total number observed haplogroups.

### Ethical statement

This study followed the guidelines adopted and approved by the institutional Ethical Review Committee at Dasman Diabetes Institute, Kuwait in accordance with the declaration of Helsinki.
